# Collectanea Medica

**Published:** 1814-04

**Authors:** 


### COLLECTANEA MEDICA,

CONSISTING OF

ANECDOTES, FACTS, EXTRACTS, ILLUSTRATIONS, QUERIES, SUGGESTIONS, &c.

RELATING TO THE

*History or the Art of Medicine, and the Auxiliary Sciences.*

### *Practice of Medicine among the Patagons.*

"AMONGST them the practice of physic is reduced into a very narrow compass, and takes in no more than vomiting and phlebotomy. These two evacuations must answer answer all varieties of cases and purposes; and where this will not do, the disease is incurable in their land. Their way of bleeding is to give a good chop with some edged tool or other in the part that is affected, be it leg, or arm, or face. Their mode of procuring a vomit is to thrust an arrow a foot and a half down the throat, which speedily produces the effect."—*Magellan's Voyage to Magellanica, &c.* 1519.

---

*Practice of Midwifery, and Management of Sick People, among the Toccoupinambaultii.*

"As touching the travail of women, I and another *Frenchman*, lodging in a certain village, about midnight heard a great outcry of a woman, and supposing she had been surprised by the cruel beast *Jan-ouare*, we arose and ran unto her, and found the woman in travail, to whom the husband performed the office of a midwife. He received the infant into his arms, cut the navel-string asunder with his teeth, but pressed down the nose (for they esteem the beauty of children to consist in the flatness of the nose). The new-born infant is presently washed, and painted by the father with colors black and red; then, not being wrapped in swaddling-clothes at all, it is put into a cotton hanging bed.

"If peradventure it happens that any of them fall sick, when the inward and familiar friend of the sick person hath declared the grieved part, one sucketh it with his mouth. Sometimes also that service is performed by certain impostors whom they call *pages*, that is physicians or surgeons. (But they are a kind of people differing from the Caraibes, whereof I have spoken before.) And they also say, that they draw out the pain, and prolong the life. They are sometimes sick of fevers, and other common diseases, but not so often as we used to be. Moreover they are troubled with a certain incurable disease which they call *pians*. This for the most part proceedeth from lust; yet I have seen the little children infected therewith, not unlike the manner of our country measles. This contagion breaketh out into pustules broader than a thumb, which overspread the whole body, and also the face itself. They never give meat to the sick unless he desire it, although he be ready to perish through hunger. Moreover, although it be the most grievous disease, they that are healthy never cease dancing, singing, and drinking, after the accustomed manner, to dull and weary the miserable sick person with the noise. Nor doth he complain, because he knoweth that he shall not prevail at all."—*Villegagnon's Voyage to South America*, 1557.

*Singular Disorder which affected the Crew of one of the Ships under Command of Sir Thomas Cavendish, in his second Voyage to Magellanica in* 1591; *written by John Lane, who was on board the Rear Admiral of Cavendish's Squadron.*

The vessels had been nearly three years in the voyage; had been subjected to various casualties; and the one in question had parted company. The men had suffered extreme hardships and severe privations, which probably laid the foundation for the complaint described in the following narrative. "Being thus at sea, when we came to *Cape Frio* the wind was contrary, so that three weeks we were grievously vexed with cross winds, and our water consuming, our hope of life was very small. Some desired to go to Baya, and to submit themselves to the Portuguese, rather than to die for thirst; but the captain, with fair persuasions, altered their purpose of yielding to the Portuguese. In this distress it pleased God to send us rain in such plenty, as that we were well watered, and in good comfort to return. But after we came near unto the sun our dried penguins began to corrupt, and there bred in them a most loathsome and ugly worm of an inch long. This worm did so mightily increase, and devour our victuals, that there was in reason no hope how we should avoid famine, but be devoured of these wicked creatures. There was nothing that they did not devour, iron only excepted: our cloaths, hats, boots, shoes, shirts, and stockings: and for the ship, they did so eat the timbers, as that we greatly feared they would undoe us by gnawing through the ship's side. Great was the care and diligence of our captain, master, and company, to consume these vermin, but the more we laboured to kill them, the more they increased, so that at the last we could not sleep for them, but they would eat our flesh, and bite like mosquitos. In this woeful case, after we had passed the equinoctial towards the north, our men began to fall sick of such a monstrous disease, as I think the like was never heard of: for in their ankles it began to swell; from thence in two days it would be in their breasts, so that they could not draw their breath; and then fall into their cods; and their cods and yard did swell most grievously, and most dreadful to behold, so that they could neither stand, lie, nor go. Whereupon our men grew big with grief. Our captain, with extreme anguish of his soul, was in such a woeful case, that he desired only a speedy end; and though he was scarce able to speak for sorrow, yet he persuaded them to patience, to give God thanks, and, like dutiful children, to accept of his chastisement. For all this, divers grew raging mad, and some died in most loathsome and furious pain. It were incredible to write our misery as it it was. There was no man in perfect health but the captain and one boy. The master, being a man of good spirit, with extreme labour bore out his grief, so that it grew not upon him. To be short, all our men died except sixteen, of which there were but five able to move. The captain was in good health; the master indifferent; Captain Cotton and myself swollen and short-winded, yet better than the rest who were sick; and one boy in health. Upon us five only the labour of the ship did stand. The captain and master, as occasion served, would take in and heave out the top-sails; the master only attended on the sprit-sail; and all of us at the capstan without sheets and tacks. In fine, our weakness and misery was so great, that we could not take in nor heave out a sail. So our top-sail and sprit-sail were torn all in pieces by the weather. The master and captain taking their turns at the helm, were mightily distressed and monstrously grieved with the woeful lamentation of our sick men." In this condition they arrived at *Bear Haven* in Ireland, where they ran the ship on shore on the 11th of June, 1593.—*Hackluyt's Collection of Voyages.*

---

[*** We intend in future to appropriate a portion of this part of our work to selections from the several Philosophical, Medical, and Surgical Journals, which of late have multiplied in so extraordinary a degree, that it is probable some of them must shortly share the fate of some of their predecessors. But as they all contain interesting matter and valuable facts, we wish to embody as much of these as possible in the Medical and Physical Journal, which is now the longest established, and contains in its numerous volumes the greatest collection of medical opinions, cases, and original dissertations, of any periodical work upon medical subjects published in Great Britain.]

*Case of Diseased Cervical Vertebræ terminating by Anchylosis. With Observations on the Treatment of Caries of the Spine; and an Outline of a Carriage, partly upon a new construction, for the use of patients laboring under that disease.* By JOHN ARMSTRONG, M. D. Sunderland.—It has been asserted by an author, whose zeal and labours I respect, that nothing remains for any future writer on the subject about to be discussed, but to give his sanction to the measures which Mr. Pott suggested.* Without presuming to throw any additional light on this matter myself, it still seems to me partly involved in obscurity, and capable of further elucidation, particularly in what regards the treatment. My object in publishing the underwritten case and observations, is to shew the great efforts which nature frequently makes toward accomplishing the cure in caries of the spine, and, at the same time, to diminish the high confidence which the writings of Mr. Pott and some other authors are calculated to inspire, respecting the efficacy of caustic issues in this disease; and if my scepticism on the latter part of the

---

* Practical Observations on the Disease of the Joints, commonly called White-swelling, &c. &c. By Bryan Crowther. London, printed for J. Callow, page 216.

subject should appear to many of too decided a character, the best apology which I am able to offer at present, is, that it has not arisen from idle speculations, but has been impressed upon me by weighty facts.

JOHN CUMMINGS, aged 11 years, was placed under my care on the 28th of July 1812. He was then extremely emaciated; had lost the use of the lower extremities and right arm; and possessed but a very slight command over the left, from the elbow downwards, just being able to raise the fore-arm feebly from the pillow, but not to grasp any thing in the hand. Though the muscles of the extremities were much wasted, they were neither loose nor flabby, like those of a truly paralytic limb; on the contrary, they felt tolerably firm, and there was considerable rigidity about the joints, particularly of the knees, so that much force was required to bend them, which, being effected, the heels were suddenly drawn to the buttocks. He complained of a fixed, and occasionally violent pain in the middle of his neck, which was always much aggravated by motion of the head or trunk in any direction. On examining the cervical vertebræ, the patient shrunk with a kind of agony when I pressed my fingers rather forcibly upon the bones; there was, however, no angular projection; but the neck seemed rather twisted towards the left side, and the head slightly inclined to the right shoulder. Here all the most material mischief existed, for the dorsal and lumbar vertebræ were apparently free from disease. The right external ear was one complete secreting surface; the glands, both of the right and left side of the neck, were enlarged and indurated. He passed his stools and urine involuntarily; the former of which were scanty, dark, and fetid. His abdomen was tense and swollen; his face pale, shrunk, and sharp; the edges of his eyelids tender and inflamed. The tongue was white, and covered with a kind of slime; and his appetite said to be keen and voracious; his pulse beat about 130 in the minute, and with more force than might have been expected from his general weakness and emaciation. He breathed in a hurried, feeble, and anxious manner; and now and then was troubled with a short dry cough. He had hectic heats and chills daily, became very feverish at nights, perspired more or less every morning, after which he obtained some short and unrefreshing sleep.

From the mother of this poor boy, the following particulars were collected respecting him. About the Christmas of 1809 he had the scarlet-fever, from which with great difficulty he recovered; and was left so much enfeebled, that nearly twenty weeks elapsed before he was able to walk out into the open air. He gradually, however, regained his strength, and, towards the close of the following year, went to attend the trap-doors in the coal-mines. This employment required but little exertion, as he had, for the most part, either to sit or stand, and, at first, it seemed to produce no material change upon his health; but at the expiration of three or four months he became dull and sluggish, began to lose his flesh, and had no relish for his usual amusements, notwithstanding his appetite remained good, and he slept tolerably well. His parents, suspecting that the coldness and dampness of the mine occasioned this change, were anxious that he should leave his occupation; but as his father was then then incapable of laboring for the support of the family, all their entreaties were unavailing. About this time he began to complain of the pain in his neck, which never left him, though he continued regularly at work until March 1812. In the end of this month, weakness, numbness, and partial loss of feeling were first noticed in his arms, which increased till the 5th of May following, when he was entirely deprived of the use of them, except the power of feebly raising the left fore-arm. On the 1st of May he was observed to trail his legs behind him, and he found himself quite incapable of walking five days afterwards. Ever since that period he has been in bed, and passed his stools and urine involuntarily, and daily become more emaciated, though his appetite has rather increased than diminished during his illness.

Not having much faith in the means commonly recommended in diseases of the spine, I determined not to order the application of caustics; still, however, I conceived that something might be done by way of assisting nature in her efforts to overcome the disease. His appetite was large, while his alvine evacuations were few and scanty; with the intention, therefore, of obtaining regular motions by the bowels, I directed that two, three, or four of the aloetic pills might be taken every night; and desired that his food might be light and nutritious, such as milk and hasty-pudding, small portions of fresh animal food, mutton-broth, and the like. All other directions that might have been given with a view of promoting an anchylosis of the diseased vertebræ of the neck, had fortunately been long anticipated by the care and watchfulness of his mother. Finding that motion gave him excessive pain in the neck, she had kept him almost constantly in the horizontal posture, though she daily cleaned his bed, changed his linen, and washed him with warm water. She was also particularly careful to have his room well ventilated, and, occasionally, when the weather was mild and dry, she carried him, placed horizontally upon pillows, into the open air, and sometimes laid him upon the grass, in a warm and sheltered place. All this was accomplished in so gentle a manner as not to occasion any increase of pain. Desiring that he might still be kept as much as possible in the horizontal position, I left my little patient, not without anxiety for his welfare. As he lived some miles distant from the place of my residence, I had not an opportunity of seeing him daily, but calling upon him occasionally in the course of the summer, had the pleasure of observing his gradual and progressive improvement. The pills were regularly continued, and produced two or three copious stools daily; his tongue became cleaner, his looks more animated, and the hectic considerably diminished; he slept better, and seldom complained of violent pain in his neck. About nine weeks after my first visit, he one day desired his mother to observe that he could move all his toes and fingers; nearly at the same time, he was now and then suddenly seized with severe pains and spasms in his limbs, and also started and talked much in his sleep. In less than a fortnight from this period, he recovered the use of both arms at once, and in two days more of the lower extremities, together with the power of retaining his stools and urine. He was able to walk pretty

 well well with the assistance of crutches, which, in a few weeks, he entirely laid aside. In the end of November the sore of the right ear was healed, and the tumefied glands on the left side of the neck began rapidly to suppurate; and a spontaneous opening taking place, there was a pretty copious discharge of healthy pus for some time. When this natural drain ceased, a seton was cut in the neck, with the design of discussing the obstructions that still existed about the right parotid and neighbouring glands; this, however, seemed to produce no material benefit, and it was afterwards dried up. The purgative pills were persevered in some time longer, and the whole of the glandular obstructions at length disappeared. It may not be superfluous to remark, that before any of the glands began to suppurate, he had regained the motion of his limbs, and he walked to the dispensary to have the seton inserted in his neck.

This poor boy was seen occasionally during his illness by John Croudace, Esq. surgeon of this place, in passing with me through the village where he resided. In the course of his illness, he was also frequently visited by John Grimshaw, of Tatfield, Esq. who first recommended him to my notice, and by whose benevolent assistance his relations were enabled to procure him many comforts.

Several months have now elapsed since he regained the use of his limbs; and he has become so vigorous, that he can walk many miles without fatigue. He has occasionally called with his mother at my house, and has been examined by several of my medical acquaintance. He says he can bear as much, or even more exercise than he could before his illness, but finds his right arm not quite so strong as the left. His head is almost immoveably fixed to the spine, with a slight inclination to the right shoulder; and he is incapable of performing the nodding and rotatory motions, so that the whole trunk seems to be thrown into action whenever he attempts to move his head forward, backward, or sideways; from which it is evident that the first and second vertebræ, to say nothing of the rest, must have formed very close adhesions about their articulations. Realdus Columbus, the successor of the celebrated Vesalius in the school of Padua, notices the case of a man in whose body he discovered, on dissection, the first cervical vertebra firmly united to the occipital bone.*

In the first paper which Mr. Pott published on caries of the spine, he states, that he had never seen the arms paralyzed by it, in whatever part situated;† and in his subsequent observations on the same subject, he mentions it as a rare occurrence.‡ If I may be allowed to judge from my own limited experience, the upper extremities are generally affected when the cervical vertebræ become diseased. In the instance above described, they were affected some weeks before the lower extremities. In another part of his work, Mr. Pott, I am persuaded, is not altogether accurate in asserting, that in naturally infirm children, the curvature of the dorsal vertebræ is always the first mark of the distemper;§ for, if I mistake not, it often happens,

* De Re Anatomica, Lib. xv. page 263.

† The Chirurgical Works of Percival Pott, F. R. S. in three volumes. London, printed for T. Lowndes, 1783. Vol. III. page 401.

‡ Pott, Vol. III. page 442. § Pott, Vol. III. page 404.

in such subjects, that either the cervical or lumbar vertebræ are first attacked; and as, in examples of this kind, the bodies of the vertebræ may become carious without a visible projection, it is the more allowable to point out an incorrect and general assertion, proceeding from so high an authority in matters of this kind. The present, however, cannot be deemed a case exactly in point, notwithstanding it occurred in a naturally weak boy, and the cervical vertebræ were diseased without an incurvation from within outward; for I conceive that, in this patient, the caries was dependent, however remotely, on the scarlet fever. If scrofula be latent in the system, no complaint so generally makes it appear, in children especially, as the malignant scarlet fever, particularly if they be exposed to cold after their recovery, whilst in an enfeebled state, as happened in this instance. Many striking occurrences of this nature were presented to me about four years ago, when the scarlet fever prevailed to an alarming extent in Sunderland and the neighbourhood. No preventives against such consequences are equal to warm clothing, occasional purging, and tepid bathing.

The kind of palsy which afflicted John Cummings, if, with propriety, it may be so called, clearly indicated, that in him the disease was altogether spinal. In the true paralysis, depending upon an affection of the brain, the muscles are soft and flabby, and the joints loose and unresisting; in him the muscles were indeed shrunk, but at the same time firm and tense, by reason of which the knees had acquired considerable rigidity, so that when the legs were stretched out in a straight position, much force was requisite to bend them; the ankle-joints were stiff, and the toes strongly turned downwards, by an apparently spasmodic action, which circumstances are characteristic of diseased vertebræ.* The long-continued pain in the neck, the agony caused by motion and pressure on the cervical vertebræ, the palsy of the upper preceding that of the lower extremities, all point out the nature and precise seat of the disease. Nor can it be urged, in objection to this opinion, that there was no incurvation of the bones of the neck, since it often happens in caries of this part of the spine, and, as Mr. Pott has himself observed, particularly of the lumbar vertebræ, that, in fact, there is no angular projection: the reason of which appears to be, that the spinous processes of the cervical are almost, and those of the lumbar vertebræ quite horizontal; whereas, the long over-lapping spinous processes of the dorsal vertebræ have a considerable obliquity downwards, and, of course, are most liable to be forced outward, by the pressure and weight of the head and trunk, when the bodies of such vertebræ become carious and approximate, on account of the destruction of the intervening cartilages.† Lastly, the fixture of the head, in this

---

* Pott, Vol. III. page 434, 435.

† Observations on Disease of the Hip-joint, &c. by the late Edward Ford, second edition. See a very sensible note on this part of the subject, page 242, 243, by Mr. Copeland, the editor of that excellent work.

case, case, and the immoveable state of all the cervical vertebræ, after the completion of the cure, leave no room for doubt upon the situation and species of the disease. Here, then, is a striking example of caries of the spine terminating favourably without issues, or drains of any kind; for it must be remembered, that the glands in the neck did not suppurate till after the boy regained the motion of his limbs, and that he walked a distance of several miles to have the seton inserted in his neck. If it be contended, that there was an almost perpetual drain of matter from the right ear, I answer, that this was so small, and so removed from the part affected, that it could have little, indeed no influence over the disease of the bones of the neck. Moreover, this discharge took place before he lost the use of his limbs, and therefore seemed to have no power in retarding, much less in arresting the progress of the complaint; in short, it was a mere secretion from the external ear, and no way concerned with the caries of the spine, except, perhaps, that it owed its origin to the same scrofulous taint of the constitution. The cure, then, of this case, was not affected by any drain, natural or artificial, but principally depended upon an effort of nature, by which, in all probability, the bodies of the bones affected were brought into mutual contact, and united firmly together, the morbid action being at the same time suspended. The aloetic pills, by keeping his bowels in a regular and rather lax state, perhaps indirectly assisted digestion and assimilation: at least their exhibition proves, that daily purgation may sometimes be used, when great emaciation exists, without prejudice, if not with advantage.

A review of the above case would certainly lead one to suppose, that rest in the horizontal posture of the body was really serviceable; and other examples of the kind have convinced me, that such a mode of treatment should generally be adopted, under similar circumstances, proper regard being paid to ventilation, cleanliness, tepid ablutions, and diet. We sometimes see children, with diseased spines, placed in an erect position, daily jolted in common carriages, or carried about in the arms of servants, without attention to the posture of their bodies; all which must necessarily tend to prevent the reunion of the separated parts, and materially impede the natural cure. The same, or similar objections may be extended to spinal machines, yet the ingenious Darwin has taken some pains to recommend them, and improve, as he supposed, their construction.* When the intervening cartilages of the vertebræ are destroyed, rest in the recumbent posture must be something like placing the ends of fractured bones opposite to each other, and retaining them in that situation. No doubt fresh air and motion would be highly desirable, by way of keeping up the vigour of the system, particularly if they both could be obtained at the same time, without agitation of the parts affected. For this purpose, an inner frame of a carriage is to

* Zoonomia, Vol. III. page 141, 142, third edition, 8vo. London, printed for J. Johnson, 1801.

be be formed into a kind of crib, and hung upon jambles, like a ship's compass: the box which contains the card, represents this crib, or place, where the patient is to be laid horizontally upon a soft mattress, and the wooden case of the compass represents another and outer frame of the carriage, which is to be suspended in the ordinary way, by easy springs. For the sake of complete security, it is evident, that the centre of gravity of that part in which the patient is to recline, must be lower than its centre of motion, after the manner of the swing-cradle. The above is a mere outline; but I hope it is drawn with sufficient distinctness to enable a good mechanician to build a carriage of much easier motion than those upon the common construction.* Whether this machine at all resembles that contrived by Larrey, the French military surgeon, for the conveyance of wounded soldiers, I have yet to learn, being, at present, only partially acquainted with his writings, through the medium of the English journals.

When the spine shrinks either to the right or left side, without an incurvation from within outward, there is, as far as I have observed, very seldom any disease in the vertebræ themselves, such affections, generally speaking, depending upon relaxation of the ligaments and soft parts. In practice this is an useful consideration, as it may save many a poor child from the pain of caustics, which, in such cases, can surely answer no good purpose. And whether caustic issues have actually the power of curing caries of the spine, it may not be improper to inquire in this place.

During the prevalence of the humoral pathology, issues were held in high estimation. The celebrated Willis compares them to trenches, cut for draining fenny land, and declares, that there were scarcely any cachectical persons living in his day, but had their skins pierced in more places than a lamprey. For paralytic affections, from whatever cause originating, he recommends issues by cautery near the shoulder-blades, and seems to have been fully aware that such complaints occasionally proceeded from derangements of the spine, but evidently mistook luxation for caries. In another part of his work, he mentions more particularly the application of the cautery for diseases of the head. "For great effects," says he, "of the brain and its meninges, in infants and children, we cut an issue on the nape of the neck; to adult or aged persons, we apply a cautery on both sides the spine, betwixt the shoulder-blades, and there we often make two issues, capable of containing many peas, with great success."†

* The hint of hanging a carriage, partly upon jambles, was given to me in conversation, by John Ness, Esq. of this place. Some time afterwards I mentioned it to John Grimshaw, Esq. an ingenious mechanician, who thought, with me, that the hint was an excellent one, and who kindly assisted me in making the above brief outline.

† The London Practice of Physic; being the Practical Part of Physic contained in the Works of the famous Dr. Willis. With the allowance of the College of Physicians. London, printed for T. Basset, 1692, page 210.

 The

The similarity of this treatment to that afterwards adopted by Mr. Pott, in palsy of the lower limbs from caries of the spine, is rather striking, though Mr. Pott appears to have been unacquainted with the writings of Willis, and derived the hint of his favourite treatment, partly from Hippocrates. The practice of making issues declined, in a great measure, with the humoral pathology; and their use is now chiefly limited to scrofulous affections of the bones and their investing ligaments and membranes, in which, however, they are at present much recommended; and though they seem to have power over diseases of the larger joints, when used at an early period, yet, I conceive, that it still remains matter of dispute, whether they have any direct influence in checking the morbid action, when caries primarily attacks the bodies of bones themselves; and as the true scrofulous caries of the spine, in the first instance, preys upon the internal parts of the vertebræ,* while the surrounding ligaments are but often very little affected,† it is doubtful whether we have not generalized too much, and attributed effects to their application, in spinal distempers, which were chiefly brought about by natural operations.

Morbid dissection shews, that white-swelling of the larger joints commences in their articular investments, and is generally attended by a certain state of inflammation;‡ which, perhaps, accounts for the good effects of topical blood-letting in cases of this kind. It appears, however, that in the first stages of caries of the spine, there is no such increase of vascularity, and that the disease at once proceeds by an absorption of bone:§ between these affections, therefore, probably some specific difference exists, greater than has been by some imagined. Be this as it may, I have, notwithstanding, witnessed beneficial results from local evacuations of blood by leeches or cupping, in the commencement and progress of caries of the spine, accompanied with violent pain, and conceive that this point deserves the consideration of future inquirers. In diseases of the hip and knee-joints, blisters, applied in rapid succession, have always seemed to me of more utility than issues by caustic; and I have had several opportunities of comparing their effects. An issue soon ceases materially to disturb the part wherein it is made; but repeated blisters keep up an almost perpetual counter-irritation, independently of the discharge which they occasion. Impartiality, however, forces me to confess, that my faith has been completely shaken, both with regard to the one and the other, as efficient agents in the cure of caries of the spine; though, as far as I have remarked, blisters, suc-

* A System of Operative Surgery, founded upon the basis of Anatomy. By Charles Bell. London, 1807. Vol. II. p. 126, 127.

† Pott, Vol. III. p. 452.

‡ A Treatise on the Morbid Affections of the Knee-joint. By James Russell, F. R. S. Edinburgh, printed for W. Laing, 1802, p. 32, 33.

§ Charles Bell, Vol. II. p. 126, 127.

cessively applied, are more powerful in alleviating the pain with which it is usually accompanied.

The great energies of nature, says an eminent author, are known to us only by their effects.* And though those effects daily take place beneath the eye of the physician, they are often attributed by him to the exterior and artificial agents which he employs. Hence, the improvement of medical science has been retarded, more or less, from the earliest ages, by recommendations of innumerable specifics; and though each successive age has given ample comments on the errors of the past, yet, even at the present enlightened period, we find, that opinions concerning some remedies are as confidently delivered to the world as before, apparently supported by testimony and fact. Some of these opinions, no doubt, are well-founded; but the greater part will, in their turn, pass away, or only appear to succeeding philosophers as the memorials of credulity. That too great credit has been given to caustic issues, in organic diseases of the spine, I strongly suspect; and I trust that I may be allowed, without the charge of presumption, to state my own experience and observations on the subject, which, if I mistake not, will throw some doubt upon the efficacy of this practice. During the last six years I have seen a very great number of sick poor. Several of those who applied to me for scrofulous complaints, I found had labored, at some former period, under affections of the spine, accompanied with palsy, for the most part of the lower limbs, from which they had slowly recovered, though, in all of them, incurvations of some of the vertebræ still remained, as marks of the previous disease. Now, Mr. Pott has satisfactorily proved, that, whenever the curvature takes place from within outward, accompanied with an useless state of the lower extremities, the bodies of the vertebræ, forming the projection, have always been carious, at some period or other; consequently, all the patients I have mentioned must have labored under this very affection of the vertebræ; yet by far the greater number of them got well without issues by caustics, setons, or any similar means, and the few who had been treated after the manner recommended by Pott, did not more speedily regain their health, nor, upon the whole, were they less deformed than those who had recovered by the efforts of nature alone. Amongst my medical friends of experience and intelligence, I find great difference of opinion upon this subject; some of them suppose that they have seen good effects from caustic issues; some doubt, and others entirely deny their efficacy. This discrepancy, at least, shows that the thing is not quite so certain as the writings of Mr. Pott are calculated to make one believe. Speaking from my own personal observation, during a term of nine years, I have never seen issues by caustic of decided unequivocal benefit; and some men, whose experience has been much longer, and on whose judg-

* Paley's Natural Theology, p. 447, the eighth edition. London, 1804.

ment I can confidently rely, have been led to form similar conclusions. Mr. Pott, however, states, in the most explicit terms, that the patients, whatever might be their age, whom he attended in the early part of the distemper, all got well; and conceives that the cure in all of them was effected by the discharge of the caustics, because, continues he, in many of them, no other means of any kind were used.* Now, without at all suspecting the accuracy of so justly-respected a character as Mr. Pott, I believe that few persons of great experience could be found in the United Kingdom, whose practice has been equally fortunate in this line. In many instances I have myself seen this complaint anticipated, as it were, by the surgeon; caustics were applied, but they did not impede its advancement. Though, as a general proposition, it may be justly said that the bones are slowly excited into disease, nevertheless striking exceptions occur in highly scrofulous habits, in whom caries makes rapid and irresistible derangements of structure. When we reflect, too, that the distemper in question is decidedly scrofulous, and often accompanied with other constitutional complaints of the same nature; that it very frequently attacks the bodies of the vertebræ, or their anterior parts, and thus affects some of the contiguous internal viscera† in its very commencement; we shall not be much disposed to wonder at its progress, and even its fatal termination, under any mode of treatment, however early adopted. These circumstances being considered, I cannot but suppose that the candid declaration of another late eminent surgeon is far more agreeable to general experience. "Whoever thinks that, in every case of incurvated spine from caries, he shall succeed by the most judicious application of the caustic, will find himself disappointed.‡" Besides, Mr. Pott, in the passage already quoted, entirely overlooks those efforts of nature, which, in many cases, complete the cure without the interference of art, and which are strikingly displayed in the most extensive affections, as the following extract will tend to illustrate, better than any thing I could advance of my own upon the subject: "In this case, there were ten diseased vertebræ, between all of which the invertebral substance was totally destroyed. Several of these were partially or completely united by anchylosis; others had suffered more considerably from the caries, great part of their substance seeming to have been absorbed, as no sensible exfoliation had taken place during the life of the patient. These bones are in some measure loose and detached from each other, yet, from their roughness, and the ragged appearance of points shooting out from some part of their surfaces, it is clear that the efforts of nature were strongly exerted towards forming the same union which had evidently taken place in others; and this would probably have been effected had it not been for circumstances which attended the management of the disease. The patient was a tender and emaciated infant, living in a

* Pott, vol. iii. p. 423, 424. † Ford, p. 245, 246.

‡ Ford, p. 245.

close close and damp place, very unfavourable for a recovery from a strumous disease. He was frequently moved and taken up from the bed; yet, notwithstanding this treatment, he lived a considerable time, and at last died of the small-pox. When he was five years of age, he was admitted at the Westminster General Dispensary, in March 1789, for a carious incurvation of the spine. It was observed that the disease had not the usual angular incurvation, which appears when a few only of the vertebræ are affected, for the whole spine was bent in the form of a bow. Issues by caustics were made near the most projecting part of the spine, but they were of no use. An abscess formed, which burst during the month of June following, and continued discharging till November, when he died of the small-pox. The disease in his back did not seem to have had any influence in the fatal termination of the variolous infection. The appearance of this disease naturally gives rise to a few reflections on the treatment of the carious spine. It exhibits an unfortunate proof of the difficulty of curing this complaint by means of art, and, at the same time, affords the consolation of showing the great efforts which nature is capable of making towards the re-union of parts separated by disease.*"

The length of time that issues require to be kept open, renders their effects extremely doubtful, Mr. Pott himself declaring, that nothing is more uncertain than the time requisite for their effecting the cure in this disease: that he has seen it completed in two or three months, and known it require two years, two thirds of which time passed before there was any visible amendment.† Now, when so much time generally elapses before any material change occurs, must it not be difficult to determine with precision whether nature or art has contributed most to the cure? Had caustics been applied in the case of Cummings, it is not impossible but that I might have been greatly deceived, and ascribed powers to them which entirely belonged to nature. All the circumstances attending the cure, in cases of diseased vertebræ, must be marked with more care and discrimination than they have hitherto been by practical writers, before their conclusions respecting caustic issues can be fairly established.

If caustic issues be at all efficacious in this disease, surely it is only by indirectly assisting the natural curative process, whatever may be their mode of operation, whether it consists in exciting some degree of counter-irritation in the parts adjacent, or, as the judicious Ford supposes, in checking an external suppuration from the progress of the caries. They cannot, I conceive, directly control the morbid action itself; and it will be allowed, on all hands, that the great work of repairing the disorganized parts, must be performed by very different instruments.

---

* Ford, page 241, 242, 243, 244. See also a most excellent plate at the end of the work, illustrative of the above case.

† Pott, vol. iii. p. 459.

Scrofula, cancer, syphilis, and scurvy, are the ordinary causes of rottenness of the bones in general; and though Van Swieten relates, in his Commentaries, that he has known the cervical vertebræ affected by lues, it is, I believe, a rare occurrence; caries of the spine almost invariably deriving its origin from scrofula alone, which, together with the structure and constitution of the parts affected, render the disease the more inaccessible to art. For syphilis and scurvy, remedies have been found out which reach and arrest them wherever situated; but for the removal of any species of scrofula or cancer, such is the imperfection of our art, no means of the kind have yet been discovered; and in both the one and the other, when they assume a tangible form, the practitioner is left either to the use of the knife or topical applications, not being able to trust to medicines administered through the medium of the system. Notwithstanding these last considerations, and the acknowledged utility of caustics in some affections of the larger joints, still the evidence of facts that have already come before me, will continue to make me doubt, that they have in reality any decided influence over caries of the spine, unless future observations on the subject should afford very opposite inferences. So strong is the conviction of their inefficiency upon my mind at present, that if a child of my own were attacked with the complaint, most assuredly they should not be applied. Occasional cupping, repeated blisters, repose in the horizontal position, aperients, and a milk diet, should form the main part of my practice in the commencement; and, if the distemper advanced under these measures, my hopes should finally rest upon those energies of nature, by which, perhaps, in every instance of the kind, the recovery is principally accomplished.—*Edin. Med. and Surg. Journal.*

---

*Memoir on the Use of the Epiglottis in Deglutition*; *presented to the* 1*st Class of the National Institute March* 22, 1813, *by* M. MAGENDIE, Professor of Anatomy and Physiology, &c.—The epiglottis, which is placed between the base of the tongue and the larynx, has hitherto been considered by anatomists as a kind of valve destined to close the glottis at the moment of swallowing, in order to prevent either solid or liquid aliments from passing into the trachea. No one doubted the fact, and this opinion consequently became general. M. Magendie was of this persuasion, but stimulated by a spirit of inquiry which ought to be possessed by every physiologist, he determined to prove the truth of the assertion by direct experiments, which had not hitherto been done.

With this view, he made an incision in the neck of a setter dog, between the thyroid cartilage and the os hyoides, drew out the epiglottis, and cut the whole of it away. When he had taken the proper precautions, the operation was very simple, and the animal appeared to suffer but little. The edges of the wound were brought together by a few stitches. In about an hour after the operation, some food was given to the dog, which he ate with avidity, as was his custom; and to his astonishment he swallowed it without any pain, pain, and with the same facility that he did before the extirpation of the epiglottis. M. Magendie thought, however, that he would find some difficulty in swallowing liquids (as they are considered more difficult to pass than solid aliments), but the animal seemed to drink without any apparent inconvenience, except that a few drops of the liquid passed through the small wound in the neck.

To determine what prevented these substances from passing into the trachea after the removal of the epiglottis, a similar incision was made in the neck of another dog, and the epiglottis drawn out, so that by fixing it by the side of the wound, he could plainly see the glottis, and its alternate movements during respiration. He then poured into the mouth of the animal a small quantity of water, which was immediately swallowed, when M. Magendie observed the glottis to be shut in an instant by the ascension of the larynx. On repeating the experiment, he noticed that it closed exactly at the instant the animal swallowed. By passing the finger into the pharynx through the wound, he could produce a movement similar to that of deglutition, which being frequently repeated, he was convinced that the glottis closed completely at the moment of the deglutition either of solids or liquids.

Conceiving that the closing of the glottis was produced by the contraction of the muscles belonging to the larynx, M. Magendie thought, that by dividing the recurrent nerves which supply these muscles, and principally the muscles of the arytenoid cartilage, he should be enabled to prove his conjecture.

After having drawn out the epiglottis of a dog as in the preceding experiment, so as to distinguish the movements of the glottis, he cut the two recurrent nerves; on producing deglutition, he found the glottis continued to shut, but, he thought, not with the same degree of exactness or rapidity.

Deeming it necessary to ascertain if the dog would swallow with difficulty without the epiglottis after the division of the nerves, he removed it totally, and closed the wound by a suture. He forgot to look at the animal for some days, at the end of which time he is certain the dog ate and drank with the utmost facility.

It appearing, from these experiments, that the closing of the glottis was produced from the influence of the superior laryngeal nerves, he determined to divide these also, to discover their particular use.

This experiment was performed on a large dog, and he saw distinctly that the constriction of the glottis was not complete. The edges closed perfectly at the anterior part, but posteriorly there was a sensible interval; the two arytenoid cartilages did not approach each other so exactly as when the laryngeal nerves were not cut. To complete the experiment, he extirpated the epiglottis, and suffered the wound to heal. When the cicatrix was complete, he was permitted to eat, and M. Magendie observed that he swallowed with considerable facility, although there was sometimes a little difficulty experienced, as was indicated by a slight cough.

In another dog he divided both the recurrent and laryngeal nerves, nerves, and found the movements of the glottis ceased: the animal in vain attempted to swallow, the glottis remained open. He then removed the epiglottis, and suffered the wound to cicatrize. Every draught of liquid produced a fit of coughing, and solids passed with great difficulty.

In another animal he divided the four laryngeal nerves only, and the utmost difficulty was experienced in swallowing; every movement of deglutition brought on a most violent fit of coughing.

He repeated these experiments on different animals,—cats, rabbits, guinea-pigs, and always with the same results. He has also examined the glottis of birds during deglutition, which acts in the same manner; and explains the absence of the epiglottis in them.

The conclusion to be drawn from these experiments is, that the epiglottis does not appear to be indispensable to prevent the food falling into the trachea during deglutition, as this is effected by the glottis instantly closing with the utmost exactness; and that the glottis performs this function chiefly through the influence of the superior laryngeal nerves.

This circumstance having led M. Magendie to doubt the statement that the laryngeal and recurrent nerves were equally distributed to the muscles of the larynx, induced him to dissect the larynx of numerous men and animals, and he found it incorrect. The recurrent nerves he found supplied only the musculi crico-arytènoidei posteriores, crico-arytènoidei latèrales, et thyro-arytènoidei. That the laryngeal nerve gave a very large ramification to the musculus arytenoideus, and the remainder was like a thread which passed between the thyroid cartilage and the thyro-arytenoid muscle, between the cartilage and the lateral crico-arytenoid muscle, and lost itself in the crico-thyroid muscle.

Sometimes this last branch, instead of descending at the back part of the thyroid cartilage, passed freely through a canal in the thick part of this cartilage, before it distributed itself to the crico-thyroid muscle. This deviation was more frequent in dogs than men.

The remaining branches of the laryngeal nerves supplied principally the muscles of the epiglottis and the mucous membrane which lined the entrance of the glottis; numerous filaments pass very near the superior edge of the musculus thyro-arytenoideus, but not one was ever observed by M. Magendie to enter the muscle.

This distribution of the nerves about the larynx corresponded with the observation of the results of his experiments previously related, and pointed out in what manner the almost complete constriction of the glottis was effected, even after the division of the recurrent nerves; as the arytenoid and crico-thyroid muscles which are concerned chiefly in the closing of the glottis, from not receiving any ramifications of these nerves, cannot be prevented from contracting by their division; and explained why the glottis remained immoveably open when the laryngeal nerves were cut.

From these facts M. Magendie has drawn the following conclusions:

1. That the epiglottis is not indispensable to deglutition.

2. That

2. That during deglutition, at the moment of the ascent of the larynx, the margins of the glottis and arytenoid cartilages approximate, and the entrance of the larynx is so completely closed as to prevent any escape into it of whatever is passing into the œsophagus.

3. That the recurrent nerve, when it has reached the larynx, gives off branches only to the posterior and the lateral crico-arytenoid, and to the thyro-arytenoid muscles, whilst the laryngeal nerve sends twigs to no other muscles (of this part) than the arytenoid and crico-thyroid. Another consequence of the attentive dissection of parts about the larynx has been to confirm M. Magendie in the belief, that the crico-thyroid muscle serves to raise its cartilage at the instant of deglutition, by which the superior edge of this cartilage is carried under the inferior edge of the thyroid cartilage.

